# Recession coverage using the modified coronally advanced tunnel and connective tissue graft with or without enamel matrix derivative: 5-year results of a randomised clinical trial

**DOI:** 10.1007/s00784-022-04691-8

**Published:** 2022-08-25

**Authors:** A. Stähli, H. Y. Duong, J. C. Imber, A. Roccuzzo, G. E. Salvi, C. Katsaros, C. A. Ramseier, A. Sculean

**Affiliations:** 1 Department of Periodontology, School of Dental Medicine, University of Bern, Freiburgstrasse 7, 3010 Bern, Switzerland; 2 Department of Orthodontics and Dentofacial Orthopedics, School of Dental Medicine, University of Bern, Freiburgstrasse 7, 3010 Bern, Switzerland

**Keywords:** Modified coronally advanced tunnel, Enamel matrix derivative, Single and multiple adjacent gingival recessions, Keratinised tissue gain

## Abstract

**Objectives:**

To evaluate the 5-year results of single and multiple recession type (RT) 1 and 2 (Miller I to III) recessions treated with the modified coronally advanced tunnel (MCAT) and connective tissue graft (CTG) with or without an enamel matrix derivative (EMD). The main outcome variable was the stability of obtained root coverage from 6 months to 5 years.

**Materials and methods:**

In 24 patients, both complete and mean root coverage (CRC and MRC) and gain of keratinised tissue (KT) were assessed at 6 months and 5 years after recession coverage by means of MCAT and CTG with or without EMD. Aesthetic outcomes after 5 years were evaluated using the root coverage aesthetic score (RES).

**Results:**

At 5 years, 24 patients with a total of 43 recessions were evaluated. Eight patients (57.14%) of the test and 6 (60.0%) of the control group showed complete root coverage. MRC revealed no statistically significant differences between the two groups, with 73.87 ± 26.83% (test) and 75.04 ± 22.06% (control), respectively. KT increased from 1.14 ± 0.57 mm to 3.07 ± 2.27 mm in the test group and from 1.24 ± 0.92 mm to 3.02 ± 1.55 mm in the control group, respectively.

**Conclusion:**

Treatment of single and multiple RT 1 and 2 recessions by means of MCAT and CTG with or without EMD yielded comparable clinical improvements which could be maintained over a period of 5 years. The additional use of EMD did not influence the clinical outcomes.

**Clinical relevance:**

The use of MCAT + CTG yielded successful coverage of single and multiple RT 1 and 2 gingival recessions, while the additional application of EMD did not seem to influence the results.

## Introduction

Gingival recessions are highly prevalent in the adult population worldwide increasing in their severity and extent with age. In a recent epidemiological study based on a large-data set from the USA, it was shown that 70.7% of the population had recessions in the aesthetic zone and 91.6% had at least one recession of ≥ 1 mm somewhere in the whole dentition [1]. Over the last decades, numerous surgical procedures have been proposed to treat gingival recessions including pedicle flaps, coronally advanced flaps (CAF) or tunnelling techniques alone or in combination with subepithelial connective tissue grafts (CTG), guided tissue regeneration (GTR), enamel matrix derivative (EMD), hyaluronic acid (HA), platelet concentrates (PRF) and acellular dermal matrix (ADM) [2–9].

The most frequently used and investigated technique for recession coverage is CAF + CTG [5, 10, 11]. The additional application of EMD has been shown to positively influence periodontal wound healing and regeneration evidenced through formation of periodontal ligament, root cementum and, to a certain extent, alveolar bone while treatment using a CTG would rather lead to a reparative healing, mainly characterised by a long junctional epithelium [12, 13].

When comparing CAF alone to CAF + EMD, clinical studies showed that the combination with EMD resulted in improved root coverage and more keratinised tissue gain [14–16]. When comparing CAF + EMD and CAF + CTG, the use of EMD yielded similar clinical results in terms of root coverage (95.1% versus 93.8%), superior results in terms of early wound healing and patient-reported outcomes and inferior results in terms of keratinised tissue gain [17]. When EMD was combined with CTG and CAF, better root coverage outcomes, higher amounts of keratinised tissue and reduced postoperative discomfort were obtained compared to CAF + CTG alone [14]. A recent systematic review on the additional benefit of EMD concluded on an advantageous effect on the recession reduction however not on the keratinised tissue gain [18].

Lately with our increasing awareness of minimally invasive surgery, tunnel procedures have come into focus of clinicians and researchers. In contrast to the original technique introduced by Raetzke who inserted a connective tissue graft in a split-thickness tunnel or at that time called “envelope”, its modification advances the whole flap complex coronally over an inserted CTG [19–23]. Among the graft-based procedures, tunnelling techniques demonstrated a greater increase of keratinised tissue and better gingival texture while CAF and modified CAF showed the highest complete root coverage percentages [24]. Several studies have shown that the modified coronally advanced tunnel (MCAT) + CTG technique results in predictable coverage of multiple adjacent gingival recessions evidenced by a mean root coverage (MRC) of 90 to 96% for (RT) 1 (i.e. previously Miller class I and II) and 83% for RT 2 (i.e. formerly Miller class III) defects [25–29].

Despite the successful outcomes reported for MCAT in treating single and multiple (RT) 1 and 2 recessions, long-term data with at least a 5-year follow-up are still scarce.

Hence, the present study reports on the 5-year follow-up of a randomised clinical trial (RCT) including patients with single and multiple RT 1 and 2 [28] (i.e. formerly Miller class I, II and III [29]) gingival recessions, treated with MCAT + CTG with or without EMD [30]. The study aimed at evaluating the 5-year stability of the obtained complete and mean root coverage (CRC, MRC), keratinised tissue width and aesthetic outcomes (RES) [31].

## Material and methods

The CONSORT statement for improving the quality of reports of parallel RCT (http://www.consort-statement.org/) was followed in the preparation of this study.

### Study design and randomisation of the original RCT

This is a 5-year follow-up of a randomised control clinical trial (trial registration number: NCT02230787; ethical approval: KEK-186–13-PPR-2015079 for the randomised clinical trial, KEK-2018–01,877 for the follow-up study). The study was performed in accordance with the Declaration of Helsinki in 1975 and revised in Tokyo in 2004. After 6 months, most of the patients were sent back to the referring dentists and received maintenance therapy in the private office. The originally 40 patients were randomly assigned to the groups using a computer-generated table and opaque envelopes during surgery after CTG harvesting.

### Surgical procedure and postoperative protocol

Before inclusion into the trial, all patients received oral hygiene instruction until plaque indices of below 20% were achieved [32]. All 40 patients were treated with the MCAT technique by the same experienced clinician (AS) as previously described [30]. In brief, following intrasulcular incisions, a mucoperiosteal tunnel flap was raised beyond the level of the mucogingival junction without touching the interdental papillae. Attaching fibres and muscles were detached from the flap using microsurgical blades and Gracey curettes. A palatal CTG was then harvested from the palate using the single incision technique. In the test group, EMD was applied onto the root surfaces and under the surrounding soft tissues after root surface conditioning with a 24% EDTA for 2 min (Straumann®, PrefGel, Straumann AG, Switzerland), followed by copious rinsing with sterile saline. Following the application of EMD, the CTG was pulled into the tunnel by means of a mattress suture and fixed at the CEJ with a sling suture. Finally, the tunnel was positioned coronally to cover the graft and the recessions by means of sling sutures. Post-surgically, patients were given analgesics for 2 to 3 days and 0.2% chlorhexidine digluconate containing mouth rinses (Chlorhexamed forte, GSK Consumer Healthcare Schweiz AG, Switzerland) during the first 2 weeks postoperatively. After 2 weeks, the sutures were removed, and the patients started mechanical plaque control by means of a soft surgical brush. Regular tooth brushing was resumed at 4 weeks postoperatively.

### Clinical assessments at 5-year follow-up

**Fig. 1:**
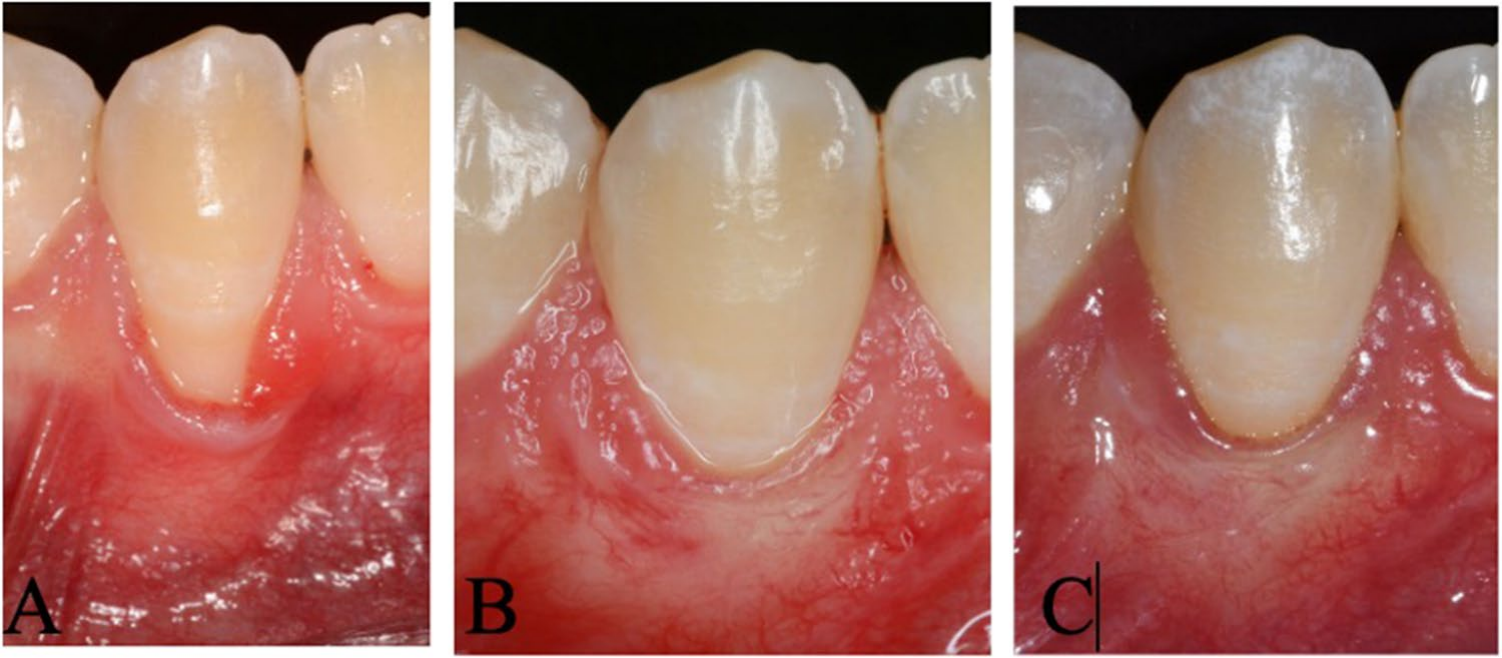
Clinical photographs of a single recession defect. Intraoral pictures before surgery (**A**), at 6 months (**B**) and 5 years (**C**). The preoperative situation shows a Miller class II or RT 1 recession defect. After 6 months, CRC was obtained with an aesthetical optimal outcome that could be maintained over 5 years

Figure 1 depicts the clinical time sequence of one single recession from baseline to the 5-year follow-up. All 40 patients were contacted and recruited for the 5-year follow-up examination for which written informed consent was obtained. The 5-year follow-up examined recession depth (RD), probing depth (PD), clinical attachment level (CAL), keratinised tissue width (KTW), CRC and MRC. All measurements were conducted by two masked clinicians (HYD and AR). The primary outcome variable was complete root coverage (CRC). Secondary outcomes were mean root coverage (MRC), clinical attachment level (CAL), probing depth (PD), width of keratinised tissue (KT) and the root coverage aesthetic score (RES) [31]. The RES represents a scoring system consisting of 5 parameters, i.e. the level of the gingival margin, marginal tissue contour (MCT), soft tissue texture (STT), mucogingival junction (MGJ) and the gingival colour. The values from the 5 parameters are summarised resulting in the final score. The highest aesthetic score is 10. Aesthetic outcomes were independently assessed on the intraoral photographs by 2 examiners (HYD and ASt). Any discrepancies were resolved by discussion between the two examiners.

### Calibration

The measurements were performed by two calibrated examiners (HYD, AR) who were masked to the group assignment. Calibration of the two examiners assessing the outcome measures was repeatedly performed on patients presenting gingival recessions. At each calibration meeting, both examiners measured recession depth, KT and PD. Calibration was accepted if more than 90% of the recordings could be reproduced within a 1.0-mm difference.

### Statistical analysis

Statistical analysis was performed using RStudio: Integrated Development Environment for R (RStudio, PBC, Boston, MA; URL http://www.rstudio.com/, accessed: 8 March 2021). Normality of distribution for quantitative variables was assessed by the Shapiro–Wilk test. Distribution of categorical variables among subgroups was assessed by Fisher’s test. The primary outcome variable was complete root coverage; secondary outcomes were mean root coverage and the amount of keratinised tissue, root coverage aesthetic score (RES), probing depth (PD) and clinical attachment level (CAL), respectively. For each clinical parameter, a patient-level analysis was performed, i.e. mean values and standard deviations were calculated for each outcome and patient, respectively. Due to the non-parametric distribution of the data, between-group comparisons including Bonferroni corrections were conducted using the Mann–Whitney *U* test for independent variables and the Friedman test for dependent variables. Significance was set at *p* < 0.05.

## Results

### Patient characteristics

A total of 24 patients were recruited from the original cohort of 40 patients. A total of three patients had moved away, and a few patients did not want to be scheduled due to the pandemic situation with COVID-19 (*n* = 3), because of pregnancy or recent childbirth (*n* = 4), lack of time (*n* = 4) or being lost (*n* = 2). A total of 43 recession defects were available for analysis comprising 14 patients with a total of exhibiting 23 recessions in the test group and 10 patients exhibiting 20 defects in the control group, respectively. In 18 patients, the recessions were localised in the mandible while in 6 of the remaining patients, the defects were located in the maxilla. A total of 12 patients were treated for multiple recessions and 17 out of 43 teeth exhibited RT2 defects (Miller class III) (Table 1). Baseline data were homogenous for both groups.

**Table 1:** Demographics of recruited patients

Patient	Tooth	Recession type defect	Gender	Age time of surgery	Smoking (yes/no)
Test group: MCAT/CTG/EMD					
1	31, 41	2, 2	f	28	No
4	32	1	f	33	No
7	31	2	f	26	No
8	22, 23, 24	1, 1, 1	m	43	Yes
13	23	1	f	43	No
14	43	2	m	56	No
15	31, 32, 33	2, 1, 2	m	28	No
17	44	1	f	28	No
18	21, 22, 23	1, 1, 1	f	45	No
19	44	1	f	32	No
20	41	1	f	23	No
22	43	1	f	22	No
23	31, 41	2, 3	f	21	Yes
25	13	1	m	24	No
Control group: MCAT/CTG					
2	31, 41	2, 2	m	22	No
3	31	2	f	34	No
5	32, 33	1, 1	f	40	No
6	41	1	f	29	No
9	31	2	f	23	No
10	31, 41	2, 2	f	28	No
11	31, 32, 33	1, 2, 2	m	29	No
12	31, 32, 41, 42	2, 2, 2, 1	m	24	No
21	13	1	m	29	No
24	21, 22,23	1, 1, 1	f	29	No

*MCAT*, modified coronally advanced tunnel; *CTG*, connective tissue graft; *EMD*, enamel matrix derivative

### Clinical outcomes

Statistically significant differences were noted for RD, KT and CAL for both groups from baseline to 5 years. Overall, CRC after 5 years was observed in 20 teeth (46.5%) belonging to 14 patients. Clinical outcomes of both groups and respective between-group differences are depicted in Tables 2 and 3.

**Table 2:** Clinical parameters in mm at baseline, 6 months and 5 years

	Test group (***n*** = 14) (MCAT + CTG + EMD)	Control group (***n*** = 10) (MCAT + CTG)	95% confidence interval	*p* value
Recession depth (RD)				
Baseline (***t***_1_)	3.79 ± 1.29	3.70 ± 1.42	− 1.500, 1.250	0.613
6 months (***t***_2_)	0.82 ± 1.02	0.69 ± 0.69	− 0.833, 0.500	0.613
5 years (***t***_3_)	0.99 ± 1.16	1.10 ± 0.93	− 0.583, 1.000	0.554
*p* value ***t***_1_ vs ***t***_2_	< 0.0001	< 0.0001		
*p* value ***t***_2_ vs ***t***_3_	> 0.999	0.9582		
*p* value ***t***_1_ vs ***t***_3_	< 0.0001	0.0003		
Keratinised tissue (KT)				
Baseline (***t***_1_)	1.14 ± 0.57	1.24 ± 0.92	− 0.500, 0.666	0.810
6 months (***t***_2_)	1.51 ± 0.66	2.25 ± 1.20	− 0.000, 1.667	0.110
5 years (***t***_3_)	3.07 ± 2.27	3.02 ± 1.55	− 1.000, 1.833	0.814
*p* value ***t***_1_ vs ***t***_2_	0.469	0.007		
*p* value ***t***_2_ vs ***t***_3_	0.013	0.999		
*p* value t_1_ vs t_3_	< 0.0001	0.005		
Clinical attachment level				
Baseline (***t***_1_)	5.84 ± 1.60	5.70 ± 1.34	− 1.750, 1.500	0.976
6 months (***t***_2_)	2.61 ± 1.03	2.32 ± 0.88	− 1.000, 0.500	0.442
5 years (***t***_3_)	2.98 ± 1.24	3.03 ± 1.02	− 0.999, 1.000	0.702
*p* value ***t***_1_ vs ***t***_2_	< 0.0001	0.0007		
*p* value ***t***_2_ vs ***t***_3_	0.77	> 0.999		
*p* value ***t***_1_ vs ***t***_3_	0.005	0.0076		
Probing depth				
Baseline (***t***_1_)	2.00 ± 0.55	2.00 ± 0	− 0.000, 0.000	> 0.999
6 months (***t***_2_)	1.85 ± 0.86	1.40 ± 0.51	− 1.000, 0.000	0.182
5 years (***t***_3_)	1.95 ± 0.83	1.93 ± 0.34	− 0.000, 0.300	0.839
*p* value ***t***_1_ vs ***t***_2_	0.999	0.172		
*p* value ***t***_2_ vs ***t***_3_	0.999	0.220		
*p* value ***t***_1_ vs ***t***_3_	0.999	0.999

**Table 3:** Comparison of the test group and the control group for variable of recession depth reduction (mm), % mean root coverage and % defects with complete root coverage 6 and 12 months after surgery

	6 months				5 years			
	Test group (MCAT + CTG + EMD)	Control group (MCAT + CTG)	95% CI	*p* value	Test group (MCAT + CTG + EMD)	Control group (MCAT + CTG)	95% CI	*p* value
Recession depth reduction	2.97 ± 1.22	3.01 ± 1.36	− 1.000, 1.500	0.837	2.79 ± 1.43	2.60 ± 1.18	− 1.167, 1.000	0.701
% mean root coverage (MRC)	80.48 ± 24.44	83.50 ± 14.85	− 13.333, 19.444	0.722	73.87 ± 26.83	75.04 ± 22.06	− 22.222, 20.833	> 0.999
Patients with complete root coverage (CRC) ≥ 1 tooth	8 (57.14%)	3 (30.0%)	n.a	n.a	8 (57.14%)	6 (60%)	n.a	n.a
Root coverage aesthetic score (RES)	n.a	n.a	n.a	n.a	8.26 ± 1.60	8.27 ± 1.54	− 1.333, 1.500	> 0.999

*n.a.*, not assessed; *CI*, confidence interval

The reduction of mean RD averaged 2.97 ± 1.22 mm and 3.01 ± 1.36 mm for the test and control group after 6 months and 2.79 ± 1.43 mm and 2.60 ± 1.18 mm after 5 years. In the test group, the mean RD decreased statistically significantly from baseline 3.79 ± 1.29 mm to 0.82 ± 1.02 mm at 6 months and to 0.99 ± 1.16 mm after 5 years, corresponding to a mean root coverage of 80.48 ± 24.44% at 6 months and 73.87 ± 26.83% after 5 years. Complete root coverage was obtained in 8 patients (57.14%) and 10 teeth (43.47%).

In the control group, the values were comparable with recession depths decreasing from 3.70 ± 1.42 to 0.69 ± 0.69 mm and 1.10 ± 0.93 mm, respectively. Mean root coverage reached 83.50 ± 14.85% after 6 months and 75.04 ± 22.06% after 5 years. Complete root coverage was achieved in 6 patients (60%) and 10 teeth (50%).

KT increased statistically significantly and comparably in both groups from baseline to the 5-year follow-up (test group: 1.14 ± 0.57 mm to 3.07 ± 2.27 mm versus control group: 1.24 ± 0.92 mm to 3.02 ± 1.55 mm).

### Root coverage aesthetic score

The mean RES was 8.26 ± 1.60 for the test and 8.27 ± 1.54 for the control group yielding no statistically significant difference between the groups. The maximal RES score was obtained in three patients and in 9 teeth of the test group as well as of the control group. Keloid formation was observed in two cases, one in each group (Table 3).

## Discussion

The present study has evaluated the 5-year results in terms of root coverage (i.e. CRC, MRC), KT and RES following treatment of single and multiple RT 1 and 2 recessions by means of MCAT and CTG with or without EMD. The results revealed that the use of MCAT and CTG resulted in successful short- and long-term outcomes in single and multiple RT 1 and 2 recessions, while the use of EMD did not seem to influence the results.

The present results provide evidence indicating that both treatment protocols resulted in predictable short- and long-term recession coverage (i.e. up to 5 years), but failed to reveal statistically significant differences in any of the investigated parameters between the groups at any timepoint.

When interpreting the results, we need to be aware that initially, this RCT was performed to specifically investigate the early wound healing events following recession coverage by means of MCAT with and without EMD. Although there is a body of evidence that EMD is able to enhance periodontal wound healing/regeneration and has been shown to result in a shorter epithelial length and higher amounts of root cementum, periodontal ligament and bone [13], the 6-month results of the present study have failed to reveal any differences in terms of inflammatory markers and clinical parameters [30]. The 5-year follow-up showed a decrease of mean root coverage (MRC) from 80.48 ± 24.44% in the test and 83.50 ± 14.85% (control) at 6 months to 73.87 ± 26.83% for the test and 75.04 ± 22.06% for the control group at 5 years. Complete root coverage (CRC) after 5 years amounted on tooth and patient level to 46.5% and 54.1%, respectively, without statistically significant differences between the groups.

When comparing the present results to those of others, our 6-month results are in line with those of a systematic review from 2018, which included 20 articles all using the tunnel technique in various combinations, and calculated a MRC of 82.75 ± 19.7% for localised and 87.87 ± 16.45% for multiple gingival recessions [6]. When analysing the results as related to MRC, the outcomes varied from 64.7 to 95.3% for connective tissue graft (CTG), from 70.5 to 91.5% for enamel matrix derivative (EMD) and from 55.9 to 95.4% for coronally advanced flap (CAF) [33].

However, consistently superior results were obtained when only RT 1 recessions were included as it was the case in the following 3 RCTs: Azaripour et al. compared CAF with MCAT, both in conjunction with CTG, and reported a MRC of 98.3% and of 97.2%, respectively, after 1 year [34]. In another study, the outcomes obtained with CAF + EMD + CTG or with CAF + CTG failed to show statistically significant differences after a follow-up period of 12 months [35]. Complete root coverage was obtained in 68% of the test group and in 52% of the control group [35]. Similarly, to the present study, a very recent RCT compared MCAT + CTG with or without EMD, using a split-mouth design. MRC reached 87.4% for the EMD-treated group and 90.9% for the control group (i.e. without EMD) with the corresponding CRC values amounting to 86.7% and 85.3%, respectively [36].

With regard to long-term results, we observed a decrease of MRC at 5 years by − 7.3% compared to the 6-month results. This might have several reasons: some patients experienced pregnancies which is correlated with increased gingival inflammation, others might have reversed to traumatic brushing habits or simply ageing might have contributed to a recession relapse. Greater a relapse in terms of MRC was reported by Zuhr and co-workers who re-examined 18 patients 5 years after treating single and multiple maxillary recessions [37]. They observed a deterioration of MRC by − 16.75% for tunnel + CTG dropping from 99.2% at 6 months to 82.2% after 5 years. However, others reported similarly excellent outcomes for mean and complete root coverage without any deterioration at 4 years following treatment of maxillary RT1 defects with either the pouch/tunnel technique or CAF, both combined with a CTG [38]. For the pouch/tunnel + CTG group, MRC measured 91.3% after 6 months and 90.1% after 4 years, with the corresponding CRC values of 89.5% and 81.3%, respectively. When interpreting these results, it has to be mentioned that discrepancies in the outcomes are likely to be related to aspects such as the type of gingival recession, their localisation (i.e. maxillary or mandibular area), root convexity or surgical technique. Indeed, in both previously mentioned studies, only maxillary and mostly RT1 defects were treated, whereas our study included a considerable number of RT2/formerly Miller class III recessions. Furthermore, most of the cases treated in the present study (i.e. 18 out of 24 patients) exhibited recessions in the lower jaw which is a technically more demanding indication. Another important aspect is the initial recession depth (RD). In our study, the mean initial RD measured 3.79 mm in the test and 3.70 mm in the control group, which is higher than in other studies [37–42].

It has to be also pointed out that so far, there is a lack of long-term studies assessing the tunnel technique + CTG with or without EMD over a follow-up period of 5 years or longer while other surgical techniques have been evaluated long-term. For example, a 12-year follow-up of a randomised controlled trial reported a reduction of MRC by 16.5% for CAF and CTG between the 6-month and the 12-year evaluation. At the re-evaluation, MRC amounted to 74.5% for CAF + CTG [39]. These results and those of others suggest that the outcomes following recession coverage have a tendency to deteriorate long-term [40, 41]. Treatment of multiple adjacent RT1 and 2 defects (Miller class I, II and III recessions) with MCAT and a porcine acellular dermal matrix showed a decrease of mean root coverage from 72.0% at 1 year to 56.7% at 4 years [41].

A high correlation between the 6-month and 3-year MRC was shown reaching 89.9% after 6 months and 91.7% after 3 years when applying CAF and a collagen matrix. The corresponding values for CAF alone were 83.7% and 82.8% [42, 43]. Interestingly, other studies observed an improvement of MRC and CRC for maxillary RT1 defects over the course of 10 years—a phenomenon known as creeping attachment [44]. However, here needs to be said, that their short-term values for MRC and CRC only reached 48.4% and 15.4% at 6 months, and increased to 71.2% and 40.0% in the following 10 years [44].

In the present study, we observed a significant gain of KT from 6 months to 5 years (i.e. mean KT increased from 1.82 ± 0.97 mm at 6 months to 3.05 ± 1.96 mm at 5 years). This finding corroborates those previously reported, which obtained a KT increase from 2.8 ± 0.5 mm after 6 months to 4.8 ± 0.7 mm after 9 years for CAF + CTG and for CAF from 3.1 ± 0.4 to 3.6 ± 0.7 mm [45]. Another group using an envelope-like coronally advanced flap recorded a mean increase of KT from baseline to the 5-year follow-up of 1.38 ± 0.9 mm [46]. Interestingly, the increase was greater at sites with initially deeper recession and lower amounts of KT [46]. Such a correlation was not observed in our 5-year follow-up study, and this might be explained by the fact that the aforementioned study only included maxillary RT1 defects, while in our study, 75% of the patients (i.e. 18 out of 24 patients) exhibited recession defects in the lower jaw with the majority of the defects classified as RT 2 defects (i.e. 10 patients). So far, it is not fully understood why and through which mechanisms keratinisation of the mucosal epithelium occurs. One possible mechanism was suggested by Ainamo et al. who observed a shift of the mucogingival junction to its original position 18 years after gingivectomies and apically repositioned flap surgeries [47, 48].

Regarding aesthetic outcomes, EMD had no effect on the 5-year RES outcome. In terms of soft tissue texture and mucogingival junction alignment, others reported on EMD yielding superior outcomes than the control group without EMD [35]. This observation appears to suggest that EMD might reduce soft tissue scarring. In our study, however, no differences in soft tissue scarring or keloid formation were observed between the groups. One potential explanation for this difference may be the different surgical techniques used (i.e. in the aforementioned study by Aydinyurt et al. [35], they used CAF and not MCAT).

In the present study, EMD did not seem to influence the clinical results, which is in contradiction with the data recently reported by Gorski and co-workers [49]. According to their regression model, EMD increased the likelihood of MRC > 85% sevenfold, of CRC 21-fold and of maximal RES tenfold after 12 months. Besides the shorter follow-up time, differences may also be due to the type of treated recessions and to their localisation (i.e. more than 90% were RT1 defects and most teeth were maxillary premolars).

The present study has some limitations. First, out of the initial cohort of 40 patients, only 24 patients could be recruited for a re-examination. This limited sample of patients might be insufficient to ensure adequate statistical power to discern intergroup differences. Nevertheless, when looking at the 6-month results with no intergroup difference, it is plausible that the long-term results do not divert between the groups. Another important aspect to be considered is the fact that most patients were sent back to their referring dentists after the 6-month follow-up. Therefore, it cannot be excluded that supportive care might have been different and some patients might have fallen back into traumatic brushing habits or less consequent self-performed oral hygiene habits.

In conclusion, the present data indicate that the use of MCAT + CTG can yield successful coverage of single and multiple RT 1 and 2 gingival recessions, while the additional application of EMD did not seem to influence the results.

## Data Availability

The data of this study are available from the corresponding author on request.
